# Community-Based Combined Lifestyle Interventions for Children with Overweight or Obesity: Exploring the Professional Teams Composition and Approach to Collaboration

**DOI:** 10.3390/children13060754

**Published:** 2026-05-29

**Authors:** Jenneke J. E. H. Saat, Elke Naumann, Merle Borremans, Willem J. J. Assendelft, Koos van der Velden, Gerdine A. J. Fransen

**Affiliations:** 1 Academic Collaborative Centre AMPHI, Department of Primary and Community Care, Radboud University Medical Centre, 6500 HB Nijmegen, The Netherlands; 2 Research Group Nutrition, Dietetics and Lifestyle, HAN University of Applied Sciences, 6525 EN Nijmegen, The Netherlands; 3 Donders Institute for Brain, Cognition and Behaviour, Radboud University Medical Centre, 6525 GD Nijmegen, The Netherlands; 4 Department of Primary and Community Care, ELG 117, Radboud University Medical Centre, 6500 HB Nijmegen, The Netherlands

**Keywords:** collaboration, teams, combined lifestyle intervention, overweight, children, descriptive cross-case comparison

## Abstract

**Background**: Community-based combined lifestyle interventions (CLIs) are used to help children with overweight or obesity achieve a healthier lifestyle. CLIs utilize the combined knowledge and expertise of professionals from a variety of disciplines. Here, we describe the composition of teams of professionals and their approach to collaboration in four community-based CLIs designed for children with overweight or obesity (focusing on children 4–12 years of age) living in the Netherlands. **Methods**: A descriptive cross-case comparison was conducted in which four community-based CLIs implemented in ten communities were conceptualized as “cases”. Quantitative data regarding the frequency of contact within the teams, topics addressed in meetings of the CLI teams, the perceived importance of other relevant disciplines in the team, and perceived satisfaction with the collaboration between professionals within the team were collected via questionnaires answered by the professionals (*n* = 82 respondents). Descriptive analyses including frequencies, percentages, and cross-case comparisons of team characteristics and collaboration were also conducted. **Results**: The CLI teams differed in composition, size, and background disciplines. The frequency of contact was higher in small teams (<6 professionals) compared to large teams. Larger teams appeared to report a lower perceived satisfaction regarding collaboration. Moreover, the role of coordinator or central healthcare provider was perceived as more important in the large teams than in the small teams. **Conclusions**: Variation was observed in professional expertise and collaboration within CLI teams. Moreover, professionals in a team should collaborate based on the local possibilities. In large teams (>6 professionals) in particular, a coordinator or trained central healthcare provider can help facilitate collaboration.

## 1. Introduction

The global prevalence of children with overweight or obesity is alarmingly high [1], as is the prevalence in the Netherlands [2]. Both overweight and obesity in children increase the risk of numerous lifestyle-related chronic diseases later in life, including type 2 diabetes, hypertension, and cardiovascular disease [3,4,5]. Moreover, overweight and obesity can reduce quality of life [6]. Supporting healthy habits should begin early in life, ideally under the age of 8, as unhealthy habits that are established in early childhood tend to persist and may therefore adversely affect long-term health [7]. In addition, preventive efforts are most effective in early life. Therefore, starting the prevention and/or treatment of overweight and obesity early in childhood is extremely important [7,8].

In the Netherlands, the prevention and treatment of children with overweight or obesity typically occurs in a primary care setting. Moreover, in many municipalities, children can participate in a community-based combined lifestyle intervention (CLI). As part of an integral network approach, CLIs focus on healthy nutrition, physical activity, and behavioral changes [9]. Although a CLI can be performed by a solo lifestyle coach, dietitian, or physical therapist [10], in practice, several professionals in various disciplines often work together, combining their knowledge and expertise in order to provide the child with appropriate, well-coordinated support and tailor-made care. However, both the composition of professionals in the CLI teams and the way in which they collaborate in performing the CLI can vary, including the disciplines involved, the frequency and structure of communication, the presence of a coordinating professional, and the degree of integration between healthcare and social care services.

A *multidisciplinary* approach refers to the involvement of professionals from different disciplines who contribute their expertise, often working in parallel. Studies have shown that a multidisciplinary approach can increase the effectiveness of interventions [11]. In contrast, an *interdisciplinary* approach involves closer integration and collaboration between professionals, including shared decision-making and alignment of goals. Professionals who work as part of an interdisciplinary team are often confronted with ideological, organizational, and relational challenges [12]. Therefore, effective collaboration requires the sharing of knowledge and information between professionals, alignment among professionals, the professionals’ ability to take different perspectives and expertise into account, and effective task management. Moreover, due to the diverse roles of multidisciplinary team members, clear coordination is needed regarding the roles and tasks both of and between the participating professionals.

On the other hand, effective collaboration increases the professionals’ collective awareness of each other’s knowledge, skills, and importance, which helps increase the team’s efficiency and quality of care [13]. Although collaborating professionals in a multidisciplinary team have been analyzed previously in the health sector [14], few studies examined collaboration among professionals who participate in lifestyle interventions designed to reduce overweight and obesity in adults [15,16,17,18,19,20]. Nevertheless, the results of these relatively few studies show that interdisciplinary confidence, role perception and role expectation are important factors in multidisciplinary approaches designed for adults with overweight or obesity. Role expectation refers to the way in which a professional believes an action should be taken, as well as the extent to which the roles and responsibilities of professionals are understood by the other members of the multidisciplinary team [16,17]. A positive attitude regarding the roles of all professionals is necessary in order to ensure that patients participate in the CLI and for effective implementation [18]. Moreover, a strong relationship and clear communication between professionals have been described as forming a good basis for the interdisciplinary treatment of adults with overweight or obesity [19]. For example, clear communication between professionals can strengthen the interdisciplinary team’s ability to work effectively as a group [17]. Previous studies (e.g., [van Rinsum et al., 2019]; [Berendsen et al., 2015]) show relatively little collaboration between key stakeholders, although the frequency of contact and familiarity with other professionals in their network were found to be essential for effective collaboration between professionals [18,20].

Thus, the composition of a CLI team—and the way in which professionals collaborate within a CLI team—can affect the team’s ability to implement the intervention in adults.

Moreover, it remains unclear to what extent findings from adult-focused CLI teams are applicable to CLI teams who target children. However, pediatric CLIs may require different forms of collaboration as children are more dependent on parents, teachers, and other caregivers. Consequently, CLIs targeting children often involve multiple stakeholders who may require complementary, yet distinct, approaches and communication strategies. Moreover, due to these differences, the disciplines of the professionals in the CLI team, the formation of the network of CLIs specific to children, and the team members’ collaboration can differ from CLIs for use in adults.

While previous studies [11,12,13,14,15,16,17,18,19] have examined multidisciplinary collaboration more broadly, empirical evidence specifically addressing CLI teams for children with overweight and obesity in practice remains limited. Moreover, although CLIs are recognized as an effective intervention method, the structure and collaboration of CLI teams for working with children with overweight and obesity have not been determined. Therefore, our aim was to determine and then describe the composition of teams of professionals and their means of collaboration in four community-based CLIs for children (focusing on children 4–12 years of age) with overweight or obesity in the Netherlands.

## 2. Materials and Methods

### 2.1. Design and Study Population

This study is informed by general concepts from interdisciplinary collaboration and team functioning, focusing on how different professional roles and coordination mechanisms are organized in practice. We conducted a descriptive cross-case comparison [21] to provide an exploratory overview of how CLI teams are composed and how collaboration is organized across different community settings. For each CLI, key characteristics (e.g., team size, disciplines involved, and perceived collaboration) were summarized. These characteristics were then compared across cases to identify variations. No inferential statistical analyses were performed as the study was exploratory in nature. Descriptive analyses including frequency, percentage, and cross-case comparisons of team characteristics and collaboration were conducted. In addition, several cases were included to capitalize on organizational variation and allow for an examination of how contextual factors can influence implementation [22].

Members of our research group connected with a contact person in each of the CLI teams. The four CLIs were implemented in the eastern part of the Netherlands. Each team of professionals performed one of the four CLIs. Here, a “CLI team” refers to a group of professionals who performed a given CLI in one specific community. For each CLI, the team’s contact person invited the entire CLI team to participate in this study.

### 2.2. Characteristics of the Four Cases

Although the specific implementation of the CLIs varied across communities (e.g., in duration, structure, and delivery), all programs were based on a CLI approach targeting nutrition, physical activity, and psychosocial well-being in children with overweight or obesity [9]. These shared core components formed the basis for comparison across cases.

The protocols for the various CLIs—and in cases in which a given CLI was implemented in several communities—differed with respect to their level of detail when describing the characteristics of the CLI (Table 1).

Each CLI was implemented in one or more communities. All CLIs in all communities included a treatment phase. The duration of the treatment phase was either six months (2 CLIs) or one year (2 CLIs). Moreover, the two CLIs with a 6-month treatment phase had either a one-year or two-year follow-up phase; the two CLIs with a one-year treatment phase did not specifically indicate the duration of their follow-up phase. All four CLIs held individual meetings (professionals met child individually) and group meetings (professionals met a group of children); the groups consisted of 5–12 children with overweight or obesity as well as the children’s family members. The study primarily focused on CLIs for children aged 4–12 years. However, some participating teams of CLI1, 2 and 3 also serve older youth (up to 19 years), which is reflected in the intervention descriptions.

CLI4 had a notably different approach than the other three CLIs; the treatment was customized to the needs and wishes of the child and family; thus, the frequency of the meetings, the disciplines involved, and the content discussed were not fixed and not described in the CLI4 protocol. In this CLI, a so-called “child health coach” (CHC) assumed the role of a central healthcare provider and served as the first point of contact for the child and their parent(s) and as the coordinator between the child’s household, the domain of health, and the social domain [23]. After assessing the child’s medical, behavioral, and social context (including family situation, lifestyle factors, and support needs), the CHC involved relevant professionals such as dietitians, physiotherapists, or social workers, depending on the identified needs of the child and family [24].

### 2.3. Data Collection

Data were collected between November 2018 and February 2019 using an online questionnaire. All professionals received this questionnaire by email from the coordinator of the CLI. Professionals who performed a CLI in several communities completed a separate questionnaire for each community. A study-specific questionnaire was developed for the purpose of this exploratory study to capture practical aspects of team composition and collaboration within CLI teams and was based on a questionnaire used in prior studies [11,18,20]. It consisted of 28 items across 3 domains (e.g., interdisciplinary collaboration, the way of collaboration, and collaboration satisfaction).

The first part of the questionnaire was designed to assess the professional’s interdisciplinary collaboration. The respondent was therefore instructed to indicate the professional(s) with whom they collaborated from a list of all team members provided by the CLI coordinator (or “Other” for any professionals not included in this list). If the respondent did not collaborate with any of the professionals in the list, they were instructed to fill in the option ‘None’.

The second part of the questionnaire contained questions regarding the way in which the professionals collaborated. These questions were based on recent studies regarding networks of prevention programs for adults with overweight or obesity [11,18,20]. The respondents were instructed to indicate their frequency of contact (daily, weekly, monthly, several times a year, yearly, or never) with the various professionals in their CLI. In addition, the respondents were instructed to indicate which topics they discussed with the other professionals, including recruiting children to participate in the CLI, coordination tasks (e.g., organizing meetings, preparing meeting agendas), process-based tasks (e.g., creating content), implementation (i.e., practical execution of the various aspects needed to perform the CLI), treatment progress, financing (i.e., calculating income and expenses), and research (e.g., investigating the effects and/or implementation of the CLI); the respondents also had the option of adding additional topics discussed. In addition, the respondents were instructed to indicate their perceived importance of the various disciplines in the CLI team (not important, fairly important, moderately important, important, or very important).

Finally, 24 statements were listed in the questionnaire regarding four dimensions of team collaboration (product, process, person, and procedure); these statements are provided in Supplementary Materials File S1. For each statement, the respondents were instructed to indicate whether they strongly disagree, disagree, neither agree nor disagree, agree, or strongly agree. The percentage of each response was then calculated for the product, process, person, and procedure evaluations. For each CLI and community, the composition and approach to collaboration (e.g., frequency of contact, topics discussed) is presented using descriptive terms, such as the frequency, and tables that show percentages.

## 3. Results

### 3.1. Cases and Participants

The four CLIs were implemented in a total of ten communities in four urban municipalities in the Netherlands. Specifically, CLI1 was implemented in one community, CLI2 in two communities, CLI3 in four communities, and CLI4 in three communities. A total of 82 professionals completed the questionnaire, with four, eight, 28, and 42 respondents for CLI1, CLI2, CLI3, and CLI4, respectfully.

### 3.2. Composition of the CLI Teams

The composition of the four CLI teams is summarized in Table 2.

The team for CLI1 consisted of a dietitian, a physical therapist, an instructor at a sports school, and a pedagogue. All four professionals were directly involved in providing the CLI to the participating children. CLI2 involved two teams consisting of a physical therapist, a dietitian working in a clinical setting, and a psychologist. Each town had its own dietitian. CLI3 was performed in four communities within the same city. In each community, the team consisted of the following professionals from six disciplines: a dietitian, a physical therapist, two sports teachers at schools, a coordinator, a primary school nurse, and a municipal policy officer. The same policy officer covered all four communities, and the policy officer and the coordinators had no contact with the children.

The coordinators performed the process-related activities and coordinated the research and financial components; by having the coordinator cover these tasks, the other professionals in the team were able to focus on the CLI.

CLI4 was performed in two communities within a medium-sized town. The professionals in the teams worked with a CHC as the central healthcare provider. The CHC was the first point of contact for the child and their parent(s).

### 3.3. Collaboration

**Figure 1:**
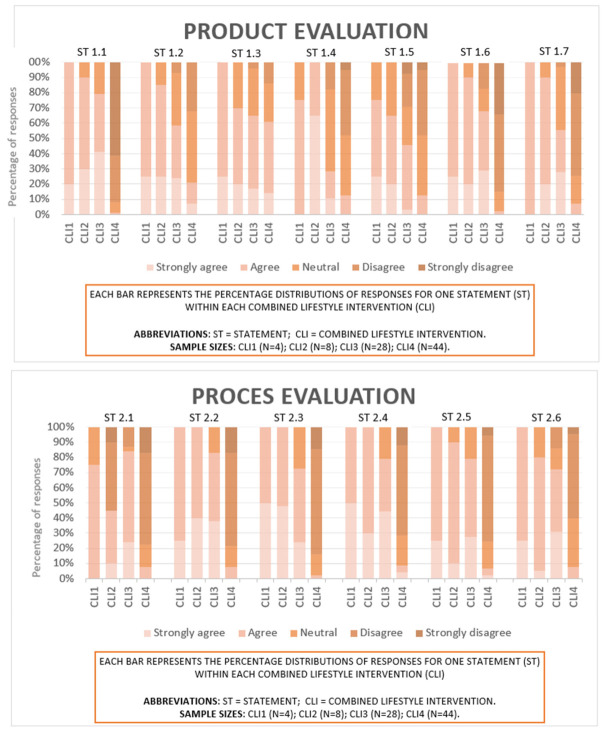

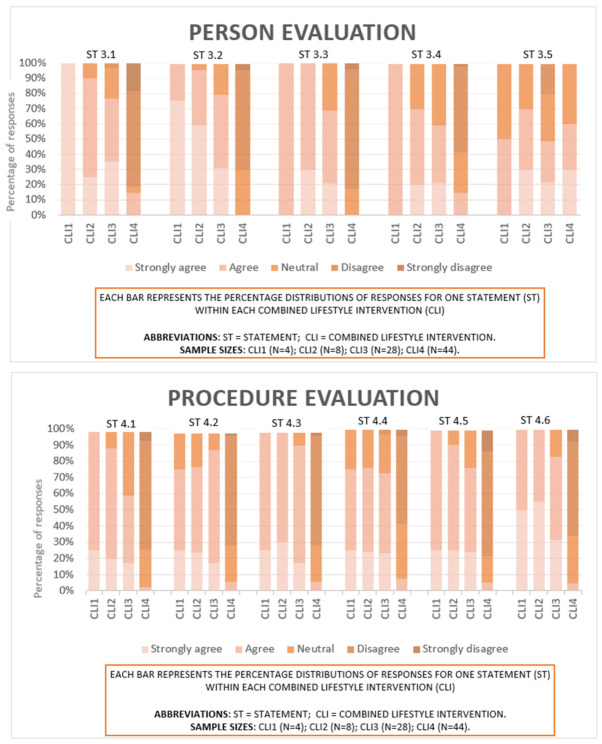
Results regarding team collaboration.

Our results regarding collaboration within the CLI teams are summarized in Figure 1 (“Results regarding team collaboration”) and are described in detail below.

We found that in some CLIs, only professionals who performed the actual content of the CLI collaborated and thus were in contact with the child and their family, while other CLIs also included team members who were only involved in the CLI during the design, organization, and evaluation phases. Generally, although some teams reported high perceived satisfaction, other teams reported that their perceived satisfaction was (very) low, with perceived satisfaction regarding the collaboration varying substantially among the teams. Larger teams (>6 professionals) appeared to report lower perceived satisfaction with the collaboration. Moreover, the professionals in the CLI teams reported the highest perceived satisfaction regarding the product (e.g., time and energy for reaching goals) and person (e.g., your role in the team is clear), in contrast to the lowest perceived satisfaction regarding the process (e.g., the composition of the CLI team is balanced, and various professionals make sufficient use of each other’s expertise) and procedure evaluation (e.g., everyone knows exactly what is expected of them).

#### 3.3.1. CLI1

##### Meeting Frequency, Topics Discussed, and Importance

The professionals in this CLI team reported that they had contact with each other on a “monthly basis” or “several times a year”. The physical therapist had the most frequent contact with the other team members. With respect to the topics discussed, the professionals contacted each other most frequently regarding the recruitment of children to participate in the CLI, followed by the implementation of the CLI. Neither financing nor research were discussed within the team. Although most topics were discussed with the team’s pedagogue, the other three professionals were involved at least several times a year. All four professionals considered their fellow professionals to be either important or very important, while the pedagogue was deemed to be “very important” by all other members.

##### Product, Process, Person, and Procedure Evaluation

The professionals in the CLI1 team were satisfied regarding the product, process, person, and procedure evaluation. In particular, they were satisfied regarding the collaboration in their team with respect to the work arrangement, which was acceptable to everyone (statement 4.1: 100% (strongly) agree or neutral), and the balanced composition of their team (statement 2.3: 100% (strongly) agree or neutral). In addition, each professional made use of the other team members’ expertise (statement 2.4: 100% (strongly) agree or neutral). Furthermore, their own role in the team was clear (statement 3.1: 100% (strongly) agree or neutral), and each professional knew exactly what was expected of them (statement 4.4: 100% (strongly) agree or neutral). Importantly, the professionals indicated that their collaboration provided sufficient motivation to continue providing the CLI (statement 1.2: 100% (strongly) agree or neutral).

#### 3.3.2. CLI2

##### Meeting Frequency, Topics Discussed, and Importance

The professionals in this CLI team indicated that they generally had contact with the other professionals on their team on a “monthly base” or “several times a year”, and their most frequent contact was with the physical therapist. They met with the dietitian “several times a year”.

The most frequently discussed topic was the recruitment of children to participate in the CLI, followed by the children’s progress. As with CLI1, the CLI2 team did not discuss financing or research. Most of the topics listed in the questionnaire were discussed with the psychologist, who was also the professional deemed to be “very important” by the other team members.

##### Product, Process, Person, and Procedure Evaluation

Most of the professionals in the CLI2 team reported being satisfied with the collaboration within their team. Each professional made use of the other members’ expertise (statement 2.4: 100% (strongly) agree or neutral), and the collaboration provided them with both inspiration and energy (statement 3.3: 100% (strongly) agree or neutral). Lastly, although it was rated the least positively by CLI2 among all four CLI teams, two-thirds of the professionals in CLI2 either agreed or strongly agreed with the statement that they were able to use their qualities within the team (statement 3.4: 66% (strongly) agree or neutral).

#### 3.3.3. CLI3

##### Meeting Frequency, Topics Discussed, and Importance

The most frequent contact for the other members of the CLI team occurred with the physical therapist and/or the sports teacher. The professionals generally contacted each other on either a weekly or monthly basis. The topics discussed at their meetings primarily concerned the recruitment of children to participate in the CLI and practical issues regarding implementing the CLI. Most of the discussions included the team’s coordinator. The dietitian and primary school nurse were frequently regarded as either important or very important by their team members, while the policy advisor of the local municipality was considered to be either not important or fairly important.

##### Product, Process, Person, and Procedure Evaluation

The professionals in this CLI team reported having the highest perceived satisfaction with the process. For example, the professionals reported a good division of roles within the team (statement 2.2: 100% (strongly) agree or neutral) and reported that their collaboration ran smoothly (statement 2.5: 100% (strongly) agree or neutral). In contrast, the professionals were least satisfied with the product evaluation compared to the other three dimensions.

Although their collaboration had clear added value and was beneficial (statement 1.1: 100% (strongly) agree or neutral), they did not discuss goals or results at their scheduled meetings (statement 1.5: 70% (strongly) agree or neutral).

#### 3.3.4. CLI4

##### Meeting Frequency, Topics Discussed, and Importance

The professionals in this CLI team generally reported that they were in contact with each other “several times a year” or “never”, and the most frequent contact (either several times a year or yearly) was with the CHC (i.e., the coordinator). In addition, the professionals reported that the most frequently discussed topic was the recruitment of children to participate in the CLI, followed by process tasks, financing, and research. Most of the topics were discussed with the CHC. Finally, the dietitians and psychologists on the team were considered by their fellow professionals to be either “not important” or “fairly important”.

##### Product, Process, Person, and Procedure Evaluation

In general, the professionals in this CLI team reported limited perceived satisfaction with the team’s collaboration. They were least satisfied with the process and procedure evaluation. For example, they reported that the various professionals in the team made no use—or insufficient use—of each other’s expertise (statement 2.4: 10% (strongly) agree or neutral); in addition, the tasks and responsibilities were not clear (statement 4.2: 95% (strongly) agree or neutral), and the professionals did not precisely know what was expected of them (statement 4.4: 10% (strongly) agree or neutral).

The professionals in CLI4 indicated they were doubtful regarding whether the time and energy it took for collaboration was worth it (statement 1.7: 10% (strongly) agree or neutral) or beneficial (statement 1.1: 10% (strongly) agree or neutral). In addition, only a few professionals indicated that the “right” partners were involved (statement 1.6: 15% (strongly) agree or neutral). Lastly, the domain “person” was not valued highly; examples included whether the professionals felt comfortable in the group (statement 3.2: 30% (strongly) agree or neutral) and whether the collaboration provided them with inspiration and energy (statement 3.3: 20% (strongly) agree).

Moreover, the likelihood of capitalizing on all of the expertise available in the team was minimized if the CHC—acting in the role of a central healthcare provider—performed the CLI or met one-on-one with the child. Compared to the other three CLI teams, this team contained the most professionals, and their perceived satisfaction was lower.

## 4. Discussion

The aim of this study was to determine the composition of four CLI teams and their approach to collaborating. Although all the teams in the study included both a dietitian and a physical therapist, the size and composition of the teams varied widely. Although the professionals in CLI1 and 2 reported high perceived satisfaction, professionals of CLI4 reported that their perceived satisfaction was (very) low, with perceived satisfaction regarding the collaboration varying substantially among the teams. Generally, the professionals in the CLI teams reported the highest satisfaction regarding the product (e.g., time and energy for reaching goals) and person (e.g., your role in the team is clear), and the lowest satisfaction regarding the process (e.g., the composition of the CLI team is balanced, and various professionals make sufficient use of each other’s expertise) and procedure evaluation (e.g., everyone knows exactly what is expected of them).

The differences in the composition of the teams can be explained—at least in part—by the lack of established guidelines and a “gold standard”. Therefore, some professionals started by considering what the ideal composition of a team—meaning that it best fits their local community—should be. They then searched for additional partners and/or professionals who were already in their existing network. Importantly, we found that the team members who reported low perceived satisfaction with respect to collaboration felt less of a connection with their fellow team members. Factors that may have contributed to this finding include the number of professionals on the team, the frequency of their meetings, and the way in which the team members understood the various professionals’ roles.

We found that the size of CLI teams in our study varied between as few as four professionals to as many as eighteen professionals. Larger teams (>6 professionals) appeared to report lower perceived satisfaction with the collaboration. This finding is consistent with previous studies [17,19], which found that having a smaller team size can improve collaboration as the professionals can contact each other more easily to discuss the treatment, with shorter lines of communication between professionals.

We also found that the various CLI teams typically met once or twice a year; moreover, the largest teams generally found this to be too infrequent, consistent with previous studies regarding CLIs. For example, some studies reported that some professionals never saw their other team members [17], reported insufficient contact with their team [20,25,26,27], or found that the professionals would have preferred to collaborate more effectively and more frequently with other professionals [28]. The relatively low meeting frequency could be explained by scheduling difficulties [17,29], some professionals not being used to networking or finding networking difficult and/or uncomfortable [28,29], a lack of financial compensation for their contribution to the team [29], and/or not working in the same building [17,29].

In our study, one professional in one of the four teams assumed the role of central healthcare provider. Beijer and colleagues [30] previously reported that centralization of one healthcare provider can be beneficial for the child and parents by serving as a confidant and providing shared decision-making regarding the treatment to be provided by one or more disciplines. In this respect, our study supports this approach and the role of a central healthcare provider.

Interestingly, Werbrouck and colleagues concluded that collaboration between disciplines is improved when the various professionals understand their fellow professionals’ roles in the team; moreover, they stressed the importance trust between professionals [19]. Based on the Dutch guideline for children with overweight, the decision to refer a child—and to which professional—is determined by the central healthcare provider [30]. One possible explanation for why the teams in our study met relatively infrequently is that one professional assumed the role of a central healthcare provider and therefore provided the treatment for the children themselves; thus, this professional did not necessarily collaborate with the other professionals in the CLI team, possibly resulting in lower perceived satisfaction with respect to team collaboration. Generally speaking, when the central healthcare provider serves as the main point of contact for the family, other professionals can become marginalized in the process. Although this was not explicitly assessed, our findings suggest that centralization and one healthcare provider—particularly in an early phase of the CLI—may not always be perceived positively by all professionals involved. Therefore, the challenge is to involve all professionals in the team as much and as early as possible, thereby maximizing the benefits for both the child and the parents.

Previous studies recognized the importance of training all team members in all disciplines in order for them to participate in a multidisciplinary weight-management program using an evidence-based approach [17,29,31,32]. In agreement with this, our findings suggest that some teams perceived potential benefits from involving professionals who were specifically trained in guiding children.

Because the role of a central healthcare provider is relatively new to the Netherlands, it may take longer for this person to become familiar with this role and receive the training needed to carry out this function [33,34].

One strength of our study is the use of a descriptive cross-case comparison approach. Studying four different CLIs for children with overweight and obesity and assessing four distinct aspects of team collaboration revealed which dimensions of collaboration were deemed to be satisfactory by the professionals.

On the other hand, a potential weakness of this study may be our sampling strategy as we instructed each CLI’s contact person to invite the other team members to participate. Thus, the size and composition of the teams varied widely, possibly due to differences between CLIs with respect to which professionals were considered to be a team member. For example, some CLIs only involved professionals who presented the actual content of the CLI and thus were in contact with the child and family, while other CLIs also included team members who were only involved in the CLI during the design, organization, and evaluation phases. This difference may have affected our results. Another question is whether certain professionals were not invited but still participated in the CLI. If not invited, these team members’ participation may have been minimal. Thus, the recruitment strategy may have introduced selection bias as the team contact person could have preferentially included more engaged or available members. This limits comparability across cases and may affect representativeness.

Given that general practitioners and policy makers can help stimulate a healthy lifestyle and help ensure that children receive appropriate healthcare, we would have expected more contact persons to invite policy makers and/or general practitioners. Nevertheless, our study illustrates how the contact person’s perception can affect which professionals they consider to be part of the team.

Another limitation of this study is that the collaboration was assessed using self-reported questionnaires, which can be susceptible to social desirability bias. As a result, the interpretation regarding professionals’ perceived satisfaction may be overstated in the reported outcomes. If so, this issue may be relevant for multiple CLIs. Moreover, the questionnaire was not formally validated, and no reliability analyses were conducted. As this study was exploratory and descriptive in nature, no causal inferences can be made.

Lastly, this study was conducted within the Dutch healthcare system, and contextual factors such as policy frameworks, reimbursement structures, and workforce organization may have influenced the organization of CLI teams. As these factors were not examined in depth, the findings should be interpreted within this context, and transferability to other settings may be limited.

## 5. Recommendations

Based on our findings, we can make some recommendations. First, professionals considering establishing a CLI for children should examine the various models regarding the team’s composition and collaboration in order to determine the best fit for their local community. Second, the team should invest in its collaboration, for example, by considering how frequently the professionals will meet, which topics they will discuss, and how to fill the role of a coordinator and/or central healthcare provider. Third, in cases in which the CLI will be performed by a central healthcare provider, training needs to be provided. The child health coach needs to be trained to organize and coordinate the child’s care, as well as to collaborate effectively with professionals in other disciplines. The professionals should also be trained to work specifically with children. Finally, the team’s composition and collaboration approach should be evaluated in order to gain valuable insights into which combination of composition and collaboration is best suited—and most meaningful—to the children and their families. The study does not explain why differences between the four CLIs occurred and further research is needed to do so. Moreover, future research should examine the extent to which our conclusions can be generalized to other settings and healthcare systems.

## 6. Conclusions

We found that CLI teams vary with respect to the professionals’ expertise. Moreover, we found that collaboration within the team is important, although this often depends on the options available in the local community. In a relatively large team (>6 professionals), a coordinator and/or trained central healthcare provider can help facilitate collaboration. Further research is needed to better understand how different aspects of a CLI team’s composition and collaboration relate to outcomes for children and their families.

In this respect, our study may support initiatives in which professionals are involved in the design, implementation, and evaluation of a CLI, thereby improving the overall approach to treating children with overweight or obesity.

## Figures and Tables

**Table 1:** Summary of the four combined lifestyle interventions (CLIs) included in the study.

	Treatment Phase	Follow-Up Phase	Frequency of Individual Meetings	Frequency of Group Meetings
CLI1	One year	Not available	Quarterly (nutrition-related)	Weekly (sports), Five times a year (nutrition-related and mental health-related)
CLI2	One year	Not available	Once per six weeks (nutrition-related and mental health-related)	Weekly (sports)
CLI3	Six months	One year	Treatment phase: every two weeks Follow-up phase: monthly	Treatment phase: every two weeks Follow-up phase: monthly
CLI4	Six months	Two years	Treatment phase: customized Follow-up phase: customized	Treatment phase: customized Follow-up phase: customized

**Table 2:** Overview of the composition of the four CLI teams showing the disciplines involved per task or area of responsibility for all four CLIs.

CLI			1	2		3				4		
Community			1A	2A	2B	3A	3B	3C	3D	4A	4B	4C
Total number of disciplines involved			4	4	4	6	6	6	6	9	9	10
Total number of professionals involved			4	4	4	7	7	7	7	12	13	17
Role	Meetings with child(ren)	Child health coach								*	*	*
		Primary care dietitian										*
		Physical therapist										*
		Sports teacher				*	*	*	*			
		Psychologist									*	
		Pedagogue										
	Policy-based	Policy advisor										*
	Coordination task	In the community								*	*	*
		Of the CLI										
	Referring	General practitioner								*	*	*
		Youth health doctor/nurse										*
		Pediatrician										
		Clinical dietitian										
		Primary school nurse										
	Indirectly involved	Preschool staff member										
		Primary school staff member										
		Pedagogue involved in youth work in community										

Level of involvement: Dark gray: certainly involved in treatment; light gray: involved in treatment if required by child health coach; white: no involvement in treatment. *: there are two professionals included.

## Data Availability

The data presented in this study are openly available at https://doi.org/10.17026/LS/TBF85P.

## Supplementary Materials

The following supporting information can be downloaded at: https://www.mdpi.com/article/10.3390/children13060754/s1, File S1. Statements regarding the four measured dimensions of team collaboration.
