# Supplementary material for: Community-Based Combined Lifestyle Interventions for Children with Overweight or Obesity: Exploring the Professional Teams Composition and Approach to Collaboration
Source: Children (Basel). 2026 May 29;13(6):754. doi: 10.3390/children13060754 (PMC13297160; doi:10.3390/children13060754)
Supplement: Supplementary file 1 [file children-13-00754-s001.zip › children-4241337-supplementary.pdf]

Additional File S1. *Statements regarding the four measured dimensions of team collaboration.*

**Product evaluation**	
1.1	The collaboration clearly provides added benefit; it is very helpful
1.2	The collaboration gives all professionals sufficient motivation to continue providing the lifestyle intervention
1.3	The team formulated the goals for the CLI together
1.4	The goals for the CLI are formulated in accordance with the SMART criteria
1.5	We are on track in terms of goals and results
1.6	The appropriate collaboration partners have a seat at the table
1.7	The time and energy required for collaboration are definitely worth it
**Process evaluation**	
2.1	There is sufficient support for the collaboration at both the managerial and operational levels of the various collaborating organizations
2.2	There is a good division of roles within the CLI team
2.3	The composition of the CLI team is balanced
2.4	The various professionals make sufficient use of each other's expertise
2.5	Mutual cooperation runs smoothly
2.6	There are few disagreements between the various professionals
**Person evaluation**	
3.1	Your own role in the team is clear
3.2	You get along well with the team
3.3	The collaboration gives you inspiration and energy
3.4	You are able to make good use of your capabilities in the CLI team
3.5	Without you personally, the project and/or collaboration would run less smoothly
**Procedure evaluation**	
4.1	Good work arrangements have been agreed upon and are acceptable to everyone
4.2	Tasks and responsibilities are clear
4.3	There is a feasible division of tasks among the professionals
4.4	Everyone knows exactly what is expected of them
4.5	There is a clear consultation structure, which contributes to the progress of the collaboration
4.6	There is sufficient contact and consultation among the professionals regarding the CLI

Possible answers: strongly disagree, disagree, neither agree nor disagree, agree, or strongly agree
CLI, combined lifestyle intervention; SMART, Specific, Measurable, Achievable, Relevant, and Time-bound.
